# Isotherm-kinetic equilibrium investigations on absorption remediation potential for COD and ammoniacal nitrogen from leachate by the utilization of paper waste sludge as an eco-friendly composite filler

**DOI:** 10.1038/s41598-024-61392-w

**Published:** 2024-05-08

**Authors:** Amir Detho, Aeslina Abdul Kadir, Hesham Hussein Rassem

**Affiliations:** 1 Faculty of Civil Engineering and Built Environment, Universiti Tun Hussein Onn Malaysia, 86400 Parit Raja, Batu Pahat, Johor Malaysia; 2 Faculty of Science, University of Hodeidah, Hodeidah, Yemen

**Keywords:** Paper waste sludge, Stabilized leachate, Leachability test, Adsorption, Environment sustainable, Environmental sciences, Engineering

## Abstract

The paper industry is a major environmental polluter due to paper waste sludge (PWS), often disposed of in hazardous ways. The techniques are employed to disposing of PWS are posing significant environmental hazards and risks to well-being. This study aims to evaluate PWS as a potential replacement for commercial adsorbents like AC and ZEO in treating stabilized leachate. Contact angle analysis of PWS was 92.60°, reveals that PWS to be hydrophobic. Batch adsorption experiments were conducted with parameters set at 200 rpm stirring speed, 120 min contact time, and pH 7. Optimal conditions for COD and NH_3_–N removal were identified at 120 min contact time, 200 rpm stirring speed, pH 7, and 2.0 g PWS ratio. Removal percentages for COD and NH_3_–N were 62% and 52%, respectively. Based on the results of the isotherm and kinetic studies, it was observed that the Langmuir and Pseudo second order (PSO) model exhibited greater suitability compared to the Freundlich and Pseudo first order (PFO) model, as indicated by higher values of R-squared (R^2^). The R-squared of Langmuir for COD and NH_3_–N were 0.9949 and 0.9919 and for Freundlich model were 0.9855 and 0.9828 respectively. Whereas the R-squared of PFO for COD and NH_3_–N were 0.9875 and 0.8883 and for PSO were 0.9987 and 0.9909 respectively.

## Introduction

Leachate from landfills is a foul-smelling, dark-black or brown liquid. It forms as a result of the decomposition processes that take place when precipitation and surface runoff penetrate solid waste accumulations. When gravitational forces are greater than the water-holding capacity of waste deposits, water percolates, causing an abundance of moisture that cannot be removed^1^. Leachate typically comes from the garbage buildup in landfills brought on by precipitation. It contains significant quantities of NH_3_–N, heavy metals, organic chloride salts, and organic and inorganic chemicals, all of which are harmful to ecosystems and animals^2^. It is essential to treat and lower the elevated levels of pollutants present in order to ensure the secure release of leachate into the environment without detrimental effects on the aquatic ecosystem. The leachate exhibits high levels of organic and inorganic pollutants, heavy metals, NH_3_–N, and a wide range of other contaminants, necessitating treatment and mitigation techniques^3^.

Water is the most valuable resource and is essential to human life. Several freshwater recovery techniques, among the most well-known are desalination and wastewater treatment. Nevertheless, they are expensive or demand a lot of energy to run. It is estimated that fresh water comprises up approximately 2.5 percent of the total water on Earth^4^.

The landfill age is a crucial factor in determining most effective treatment technique for leachate. It is observed that leachate generated during the initial stages of the landfills life comparatively easier to treat compared to mature or older leachate^5,6^. Leachate with a greater biodegradability ratio is frequently treated using biological techniques. However, these techniques might not be as effective in treating mature leachate, which predominately consists of resistant substances and chemicals like NH_3_–N^7^.

The author concludes that date palm fibre filters were used to treat greywater efficiently. The results show a 15% drop in turbidity and a substantial 15% decrease in TDS and conductivity. The pH level differed depending on the surroundings; it increased in industrial and commercial regions and decreased in domestic areas. This study aims to mitigate the scarcity of water and promote efficient water conservation techniques in mosques across Riyadh^8,9^.

The author, optimised coagulation/flocculation technique for slaughter house wastewater treatment using Poly Aluminium Chloride (PAC). The results demonstrate the exceptional effectiveness of PAC, with removal rates for COD, TSS and TN are 74.6%, 82.0% and 89.1%^10,11^.

The biodegradation rate and reduction of organic matter reduces when NH_3_–N concentration rises and landfills move towards the stabilization phase. As a result, biological mechanisms lose some of their efficiency over time. For stabilizing leachate in these circumstances, a physicochemical method may be recommended^12^. Adsorption is a widely utilized physical–chemical method for the elimination of persistent organic constituents from landfill leachate. Adsorption essentially entails the transition of chemicals from a liquid to a solid form, followed by physiochemical processes. A variety of adsorbent substances, including activated carbon (AC), activated alumina, zeolite, as well as less expensive alternatives including limestone, peat and clay have been studied for adsorption-based wastewater treatment. However, AC has garnered a lot of attention in recent years for removal of contamination from the leachate. This is due to its physical characteristics, like a structure with tiny pores, a significant surface area, and the ability to react with substances on its surface^13^.

In this study, the author prepared MgFe_2_O_4_ and AC composites produced from orange peel using a hydrothermal process and characterized them comprehensively. MF45-AC exhibited outstanding electrochemical behaviour with a specific capacitance of 870 F·g^−1^ at 1.0 A g^−1^ and excellent cycling stability, retaining 95.1% of its initial capacitance after 5000 cycles. These findings highlight the potential of MF-AC composites for enhancing supercapacitor performance and energy density^14^.

Industrial wastewater is a serious problem because of its pollution and water consumption. Coagulation/Flocculation is a cost-effective treatment method. The use of natural coagulants and flocculants (NC/Fs) as beneficial, long-term treatments for industrial wastewater. The author emphasizes the significance of reusing waste materials as a renewable natural resource and the effectiveness of M-NC/Fs in the treatment of industrial wastewater. In addition, the author suggests employing M-NC/Fs in full scale treatment system to effectively treat wastewater^15,16^.

Thus, Alternative materials are required to serve as adsorbents for organic substances and ion elements due to the high cost connected with their utilisation. The substantial amount of paper consumption leads to the generation of significant waste, with approximately 50–60% of this waste being recycled. PWS is a byproduct formed during the production of recycled paper, often discarded as waste from industry^17,18^. The rapid economic development and population growth in Malaysia have led to a huge increase in waste generation. Waste generation in Malaysia significantly increased between 2001 and 2005, rising from roughly 16,200–19,100 ton/day. This represents 0.8 kilogramme of waste produced per person per day on an average. In Malaysia, the paper industry alone is responsible for around 30% of trash production, and this percentage is rising at a pace of roughly 4% per year^19^. The waste referred to as PWS poses challenges in terms of disposal due to its costliness and potential environmental harm associated with landfilling or incineration methods.

PWS is generated during the production of paper using water and chemicals, which contaminates water and releases greenhouse gases into the environment. When sludge is not adequately handled and processed, it can cause a variety of environmental problems, including groundwater contamination, soil and water contamination, and methane emissions from landfills. These concerns can be fixed, by using advanced treatment methods that minimize the use of chemicals and improve the efficacy of water treatment. Examining the potential usage of sludge, such as composting or energy generation, may mitigate the material's detrimental effects on the environment and promote the reuse of resources. Overall, proactive measures and sustainable practices are essential for reducing the environmental footprint of paper production and waste managing^20^.

This study investigates, PWS effectiveness as an alternate adsorbent for AC due to its reduced cost, increasing need, and scarce resources. The primary objective is to generate an adsorbent by determining the optimal ratio of PWS. The main conclusions of this work on identifying the best dosage of PWS to achieve the highest COD and NH_3_–N reduction from leachate at their highest rates.

## Materials and method

### Leachate sampling

For this research, the leachate utilized in the research was composed from the Simpang Renggam municipal landfill (SRL) site in Johore, Malaysia. The sampling location is situated within the Kluang district of Johore, with geographical coordinates of approximately N 1° 53″ 41.64″, E 103° 22″ 34.68″. High-density (HDPE) plastic bottles that were clean and held 30 L were used to collect samples of the leachate. Before starting the experiments, these specimens were taken to the research lab and maintained in a chiller at 4 °C to preserve their characteristics and reduce any further variations. The leachate sample characterization was conducted within 24 h following the standardized approach outlined in^21^. All of the substances utilized for the analysis were of analytical grade quality^22^.

### PWS preparing method

The paper industry's PWS is one of its byproducts. The PWS samples were acquired from a paper factory situated in eastern region of Peninsular Malaysia. To get rid of any moisture content, the PWS samples were subjected to a drying process in an oven for 24 h at a temperature of 150 °C. The PWS were pulverized into 150 µm particle sizing using a ball mill. The pulverized material underwent a sieving process to obtain particles within the size range of (≥ 75 μm to retained ≤ 150 μm)^22^.

### Batch study experiment

A set of nine 250 mL flasks were used for an experiment. Each flask containing a 100 mL of leachate sample and 4.0 g of the adsorbent material. The selected adsorbent amount was based on previous studies that suggested it as an optimal quantity for achieving higher removal of NH_3_–N and COD in this particular research investigation^22^. The use of the low-cost adsorbent (PWS) in this study provides an alternate method for replacing some of the conventional materials. To find the best alternate solution, previous researchers have thoroughly explored various ratios and varied amounts of the adsorbent by weight [12; 22]. The batch-scale studies were carried out in a controlled environment, which included a temperature of 4 °C, an ideal stirring speed of 200 rpm, a shaking period of 120 min, and a pH7^23–27^. Adsorption isotherm experiment was conducted, to access the applicability of various models in the reaction mixture, which contained 100 mL of leachate water with varied concentration^22^. The best optimal dosage of PWS for effectively removing COD and NH_3_–N from the leachate solution were chosen as the best choice.

### Analysis of leachate

The COD concentration was determined using the 5220 D-closed reflux colorimetric technique, and the Nessler technique (HACH 8038) using HACH DR6000 spectrophotometer was used to determined NH_3_–N. All experimental work and the instruments used in this study were conducted at the Water & Environmental Engineering Laboratory, Faculty of Civil Engineering and Built Environment, Universiti Tun Hussein Onn Malaysia (UTHM), Malaysia. All of these techniques were performed in triplicate in according the standardized practice described in^21,22^.

### Ethics approval

This article does not contain any studies with human participants or animals performed by any of the authors.

## Results and discussion

### Leachate characterization

The SRL site provided the fresh leachate samples, which were manually collected and kept in a 30-L (HDPE) plastic container. Table 1 presents the characteristics of the unrefined leachate. The major characteristics of interest in this study are COD and NH_3_–N and their elimination is its main goal. The results of the investigation showed that the NH_3_–N content was 2040 mg/L. While the BOD_5_ and COD concentration was measured at 380 mg/L and 2993 mg/L. Additionally, BOD_5_/COD ratio was determined to be 0.14. Leachate with a greater biodegradability ratio is frequently treated using biological techniques. Biological mechanisms lose some of their efficiency over time. However, these techniques might not be as effective in treating leachate. In our study, biodegradability ratio was found at range of 0.14 in batch system, in these circumstances, adsorption is a widely utilized physical–chemical method for the elimination of persistent organic constituents from landfill leachate^28^. The pH and NH_3_–N levels of the stabilized (old) leachate are relatively high, with values < 7.5 and above > 400 mg/L. However, COD concentration and BOD_5_/COD ratio are comparatively lower, measuring below 3000 mg/L and 0.1, respectively^29^.

**Table 1:** The properties of fresh leachate from the SRL site^30^.

Parameters	Values	Average	Standard deviation
pH	8.36	8.33	0.05
SS (mgL^−1^)	215	165.50	70
NH_3_–N (mgL^−1^)	2040	1940	141.42
COD (mgL^−1^)	2993	2921.50	101.12
BOD_5_ (mgL^−1^)	380	310	98.99
BOD_5_/COD	0.14	0.11	0.04
Fe (mgL^−1^)	8.97	7.21	2.49
Color (Pt–Co)	4749	4400	493.56
Heavy metal (mgL^−1^)	Low	Low	Low

### Leachability test of PWS

The findings of the PWS leachability tests performance are presented Table 2 which reveals that the values are within the acceptable limits according to the standard. Table 2, concentrations of metals dissolved in solution from the TCLP test are higher than expected due to heavy metals are more readily released in acidic conditions^31^.

**Table 2:** PWS Leaching test findings in accordance with TCLP procedure.

Metal	Standard limit (mg/L)	Concentration (mg/L)
Cd	0.025	< 0.01
Ni	0.040	0.02
Zn	0.050	0.02
Cr	0.100	0.04
Cu	0.025	0.019
Pb	0.100	< 0.03

### Analysis of contact angle and measurement

**Figure 1:**
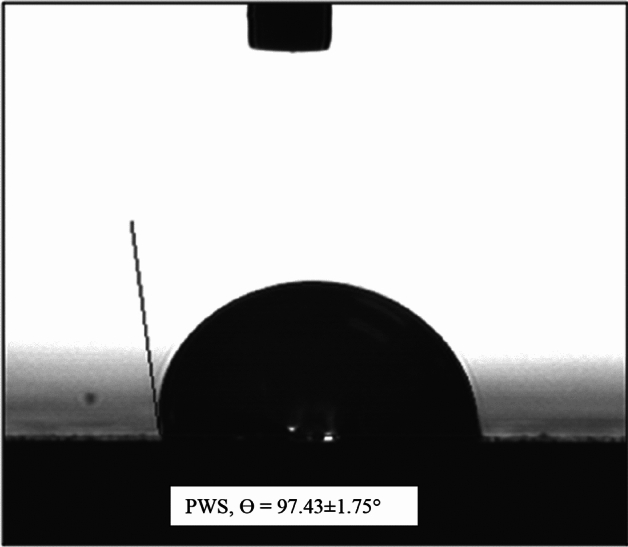
Water contact angle of PWS.

The media were classified as either hydrophobic or hydrophilic. In order to identify the hydrophobicity or hydrophilicity. The contact angle characterization of the PWS was attained using pendant drop contact angle (VCA-Optima) experiment. The contact angle analysis demonstrated that the contact angle of PWS were 97.43° respectively, indicated that PWS were hydrophobic materials as depicted in Fig. 1.

### Surface physical morphology

**Figure 2:**
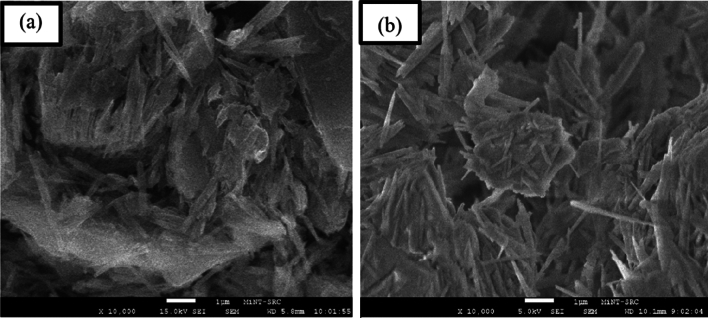
Surface morphology images of the PWS (**a**) before-adsorption and (**b**) after-adsorption.

Surface morphology images of the PWS are exhibit in Fig. 2a and b which illustrate the morphological characteristics. The scanning electron microscope (SEM) image of the PWS displays the existence of significant particles that seemed to have formed through the aggregation of multiple flaky particles stacked together, as observed in the study conducted by^32^. The SEM image reveals that the PWS exhibits distinctive hexagonal platelet shapes with a porous surface structure, which is consistent with the findings stated by^33^.

In contrast, Fig. 2b demonstrates that following the adsorption process, PWS particles have formed agglomerates by the stacking of multiple flaky particles together, influenced by the adsorption process, as described in the study by^32^.

### Transformation infrared spectroscopy analysis

**Figure 3:**
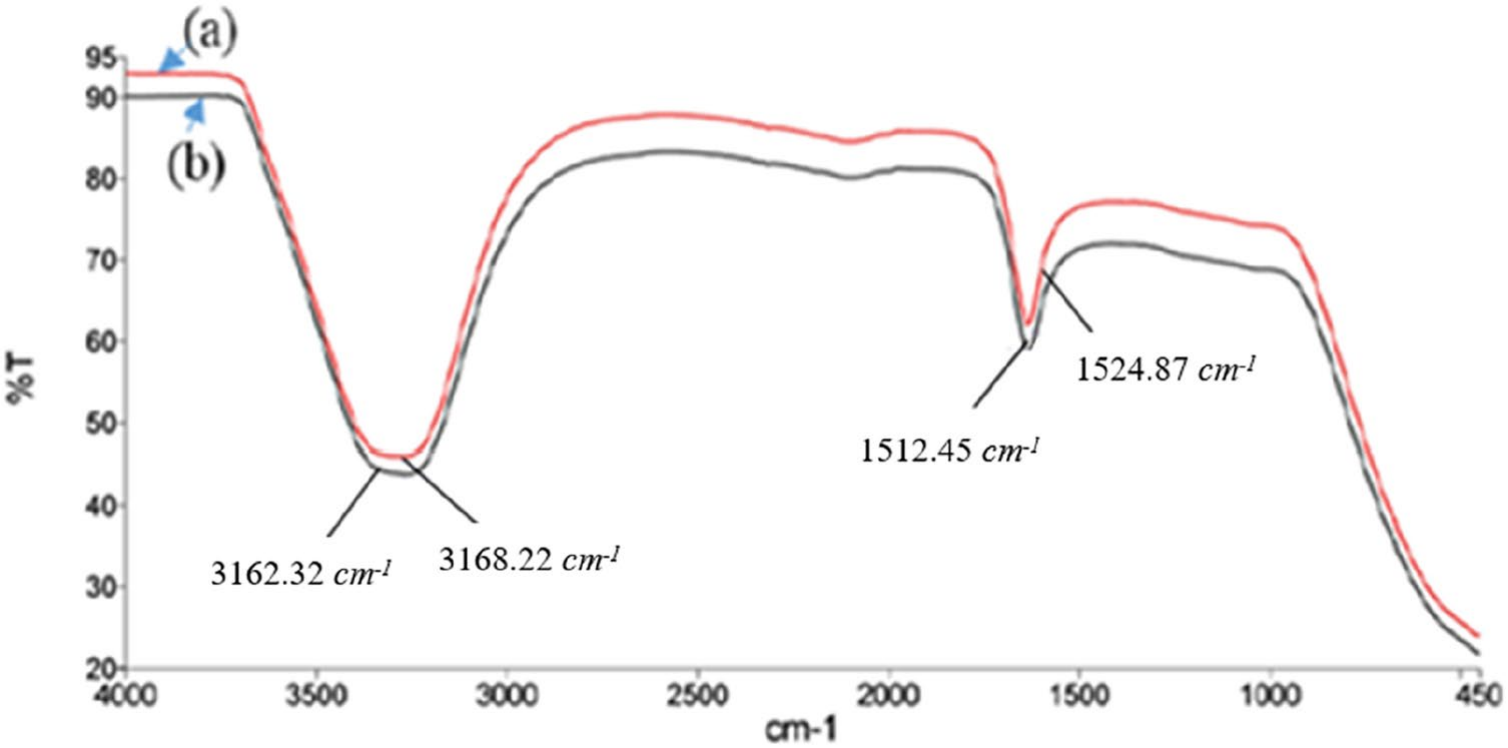
FTIR of PWS (**a**) before-treatment and (**b**) after-treatment.

In Fig. 3, FTIR spectra display the spectral data for PWS before and after an adsorption procedure. The spectra span the 400–4000 cm^−1^ wavenumber range. In both the untreated and treated samples, there is a significant peak observed in the spectra at wavenumbers 3162.32–3168.22 cm^−1^. This peak signifies the presence of polymeric associations involving hydroxyl groups and the stretching of bonded OH groups. According to studies, the C–C and C=C bonds are responsible for the spectrum of absorption bands observed between 1524.87 cm^−1^ and 1512.45 cm^−1^. The differences between the before-treatment and after-treatment states could be attributed to variations in the adsorbent, likely resulting from the incorporation of solutes into intermediate layer and voids from the water-based solution^34,35^.

### PWS dosage effect on COD and NH_3_–N

**Figure 4:**
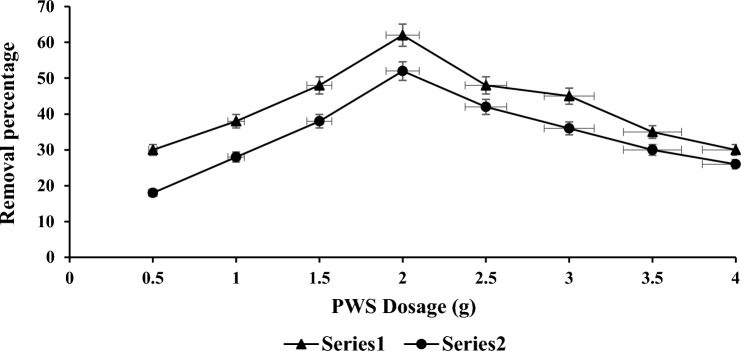
Optimal PWS dosage for COD and NH_3_–N reduction.

The optimal PWS dosage was determined by the lowest amount of PWS that removed the COD and NH_3_–N. Figure 4 illustrated that the optimal dosage was attained at (2.0 g of PWS) with highest reduction of COD is 62%. For optimal removal percentage of COD, the obtained adsorbent ratio considers as best ratio of PWS. The optimal ratio of PWS for NH_3_–N was attained at (2.0 g of PWS) with highest reduction was attained at 52%. For optimal removal percentage of NH_3_–N, the obtained adsorbent ratio considers as best ratio of PWS. Figure 4 indicates that the removal percentage of COD increases with varying ratio of PWS, because PWS is most efficient adsorbent for removing organic compounds^36^. The finding results revealed that optimal adsorbent mixing ratio at 2.0 g which results in best removal percentage for COD and NH_3_–N. The optimal adsorbent (PWS) was chosen at 2.0 g in order to significant variance in removal percentage of COD and NH_3_–N (62% and 52%).

### Effect of stirring speed on COD and NH_3_–N

**Figure 5:**
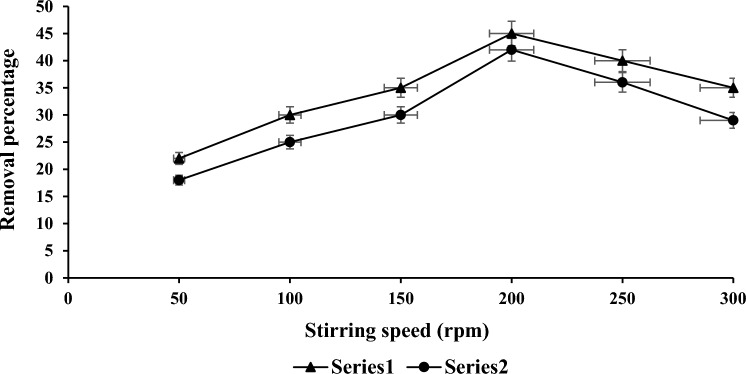
Maximum speed interval for COD and NH_3_–N reduction.

Figure 5 depicts the reduction of COD and NH_3_–N for samples that were treated with leftover PWS. The samples were shaken at several stirring speeds ranging from 50 to 300 rpm in order to determine the appropriate shaking speed. The best optimal condition of reducing the both parameters were identified at stirring speed of 200 rpm with reduction of 45% and 42%. The desired COD and NH_3_–N reduction was achieved under favourable conditions, specifically at a mixing ratio of 2.0 g, and a stirring speed of 200 rpm. The optimum reduction outcome obtained from these conditions was selected for subsequent experiments.

### Shaking time effect on COD and NH_3_–N

**Figure 6:**
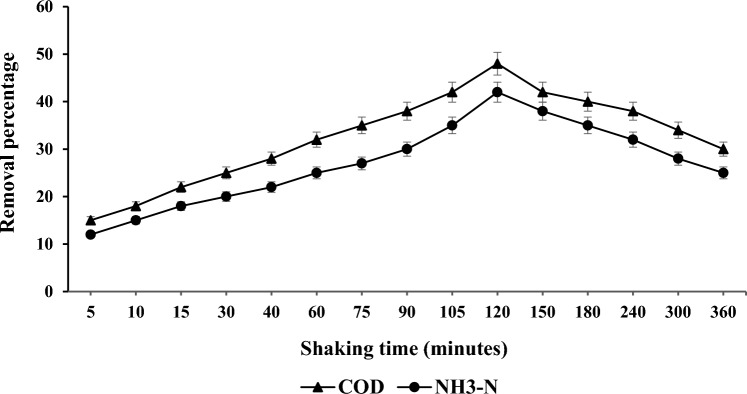
Maximum time interval for COD and NH_3_–N reduction.

It is important to determine the optimum time for the reduction of these substances. In the experiment, the shaking time was varied from (5–360) minutes as depicted in Fig. 6 while the adsorbent dosage was kept constant at 2.0 g. The optimal initial conditions for reducing the two parameters, COD and NH_3_–N, were attained after 120 min, resulting in a reduction of 48% and 42%. The desired COD and NH_3_–N reduction was achieved under favourable conditions, specifically at a mixing ratio of 2.0 g, a stirring speed of 200 rpm, and a shaking time of 120 min. The optimal reduction outcome obtained from these conditions was selected for subsequent experiments.

### pH effect on COD and NH_3_–N

**Figure 7:**
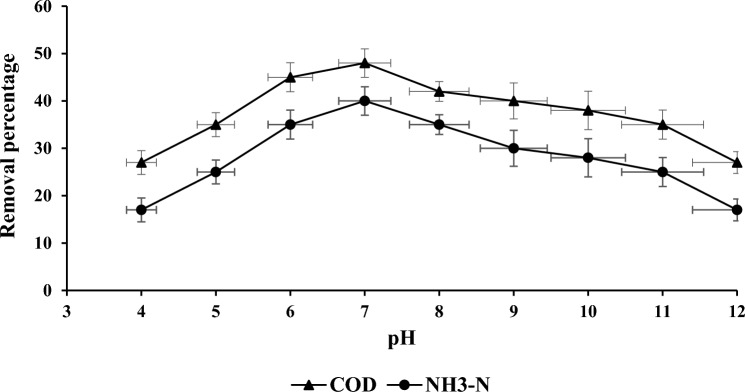
pH maximum for COD and NH_3_–N reduction.

Figure 7 illustrated the COD and NH_3_–N percentage reduction in samples treated with PWS. In order to determine the ideal pH for attaining best reduction, the samples were shaken at a range of pH values from 4 to 12. The most promising condition for reducing COD and NH_3_–N was attained at a level of pH7, resulting in both the parameter values were reduced by 48% and 40%. The desired COD and NH_3_–N reduction was obtained by utilizing a mixing ratio of 2.0 g, a stirring speed of 200 rpm, a shaking time of 120 min, and the specified pH level.

### Comparison PWS performance to other reported adsorbents

In this study, the utilization of PWS is considered as an innovation that aiming to produce a cost-effective adsorbent that replaces traditional materials like AC and ZEO. This study mainly examines and compares the properties of PWS adsorbent with other researchers' findings. The analysis indicates that PWS performance better compared with other researchers' findings. A comparison of the PWS adsorbent's removal is also shown in Table 3. Comparing PWS to different adsorbent materials, analysis shows that PWS has better removal for both COD and NH_3_–N.

**Table 3:** Comparison PWS performance to other reported adsorbents.

References	Types of effluent	Absorbent materials	Factor	Reduction
^37^	Lake wastewater	Green mussel Shell	NH_4_^+^	31.28%
			COD	44.45%
^38^	Leachate	Cockel Shell	COD	55%
^23^	Leachate	Activated carbon and green mussel	COD	83%
			NH_3_–N	63%
^39^	River water	Cockel Shell	COD	38.8%
Present study	Leachate	Paper waste sludge	COD	62%
			NH_3_–N	52%

### Isotherm adsorption analysis

According to the Langmuir, maximum adsorption takes place when a monolayer of solvent molecule covers the adsorbent surface. The equation for the Langmuir model is provided in reference^40^.

1 $${q}_{e}= \frac{Qb{C}_{e}}{1+b{C}_{e}}$$

For Langmuir constant, ${C}_{e}$ denotes the adsorbate equilibrium concentration (mg g^−1^); whereas ${q}_{e}$ denotes equilibrium adsorption (mg g^−1^). The $Q$ denotes adsorption maximum capacity (mg g^−1^) and denotes adsorption energy (L mg^−1^).

The Freundlich model is unique because it takes into account the heterogeneity of energies involved in the sorption process. The Freundlich model takes into account for variations in sorption, in contrast to the Langmuir model, where the energies change depending on how frequently the surface is covered. The equation for the Freundlich model can be found in reference^41^.

2 $${q}_{e}= {K}_{f}. {C}_{e}^{1/n}$$

**Figure 8:**
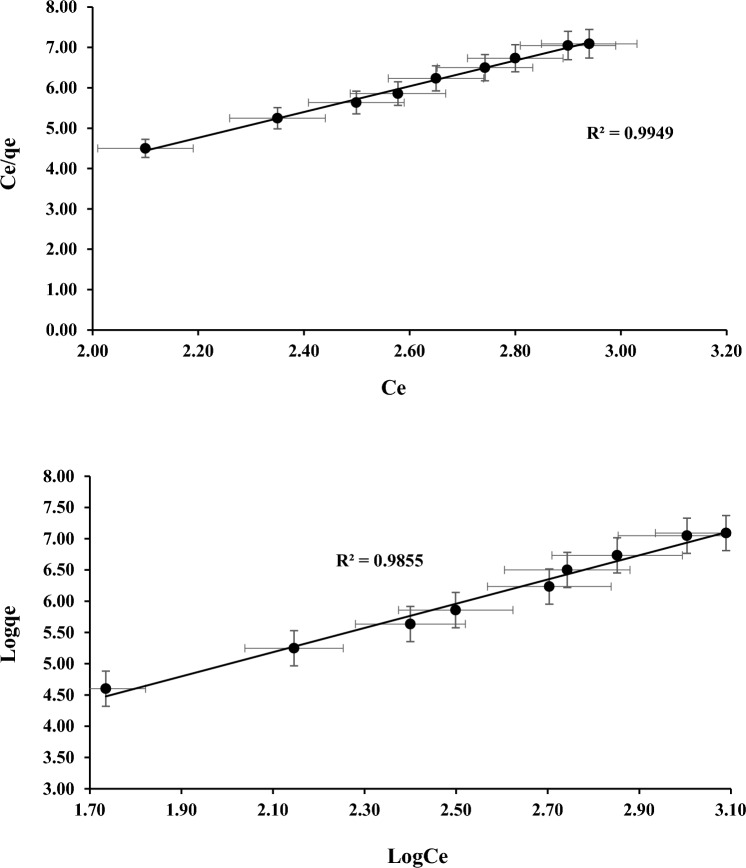
Sorption for COD (**a**) Langmuir and (**b**) Freundlich.

**Figure 9:**
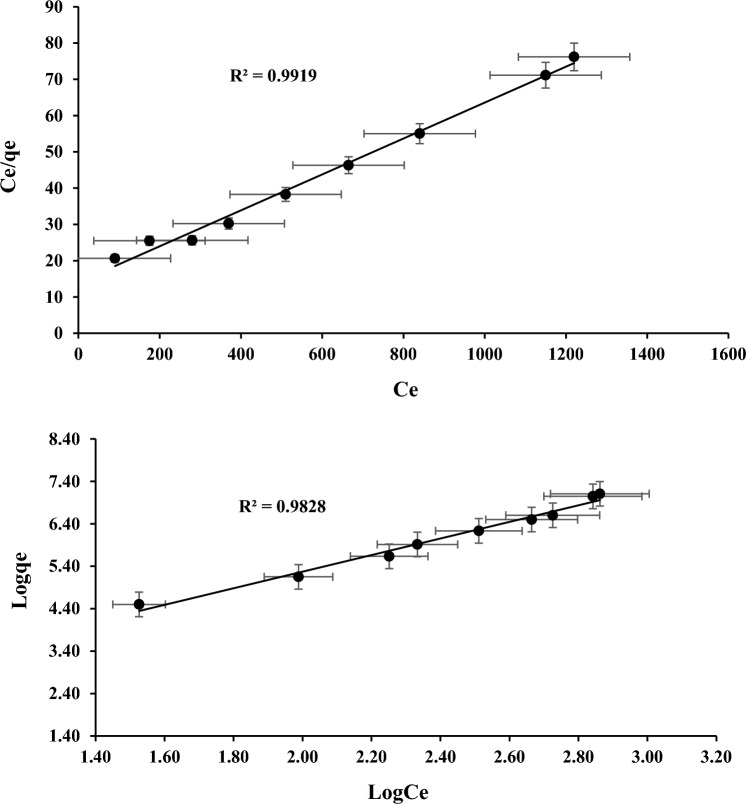
Sorption for NH_3_–N (**a**) Langmuir and (**b**) Freundlich.

For Freundlich constant, ${K}_{F}$ denote sorption capacity and $b$ denote sorption intensity. According to the experimental results for reducing percentage of COD and NH_3_–N as shown in the Figs. 8 and 9. It can be concluded that the Freundlich is less appropriate than the Langmuir. These findings suggest that the adsorption process can be precisely captured by a mono-layer coverage of both parameters on the surface of the adsorbents.

### Kinetic study adsorption analysis

Adsorption kinetics deals with the rate at which sorption occurs, where the rate is shown by the change in parameter over a predetermined time period. The adsorption kinetics analysis offers insights into the extent of absorption occurring on the surface of the adsorbent material at a given interaction time during equilibrium. In order to assess the rate of sorption, two models were employed; the PFO and PSO kinetic developed by^42^.

**Figure 10:**
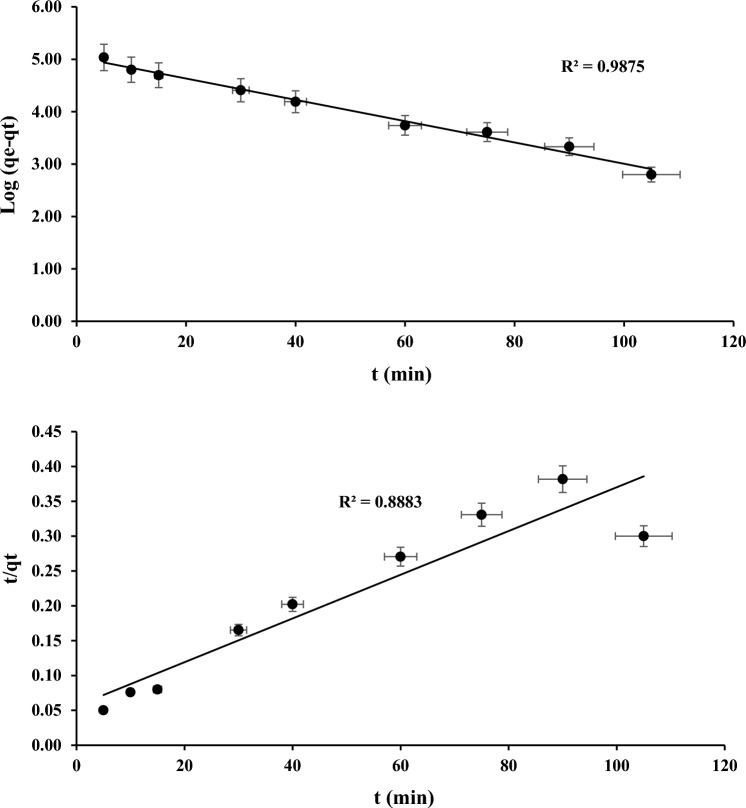
Linear graphs for the PFO of COD and NH_3_–N against a PWS.

**Figure 11:**
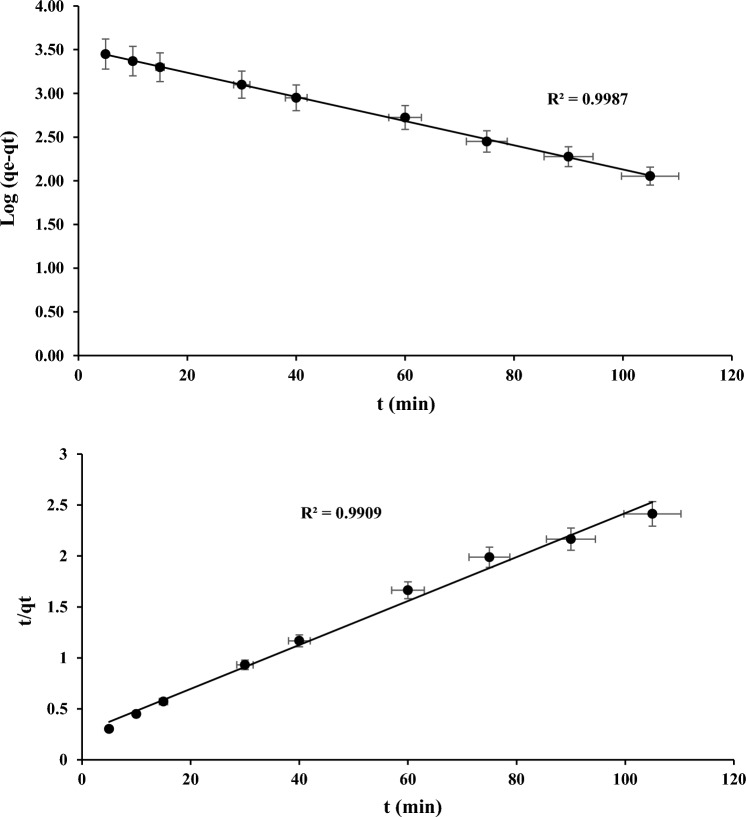
Linear graphs for the PSO of COD and NH_3_–N against a PWS.

Figures 10 and 11 depicts linear graphical representations for the adsorption of COD and NH_3_–N on PWS using PFO and PSO kinetic models, respectively. The lower R-squared (R^2^) attained for PFO shows PWS that the COD and NH_3_–N adsorption is not accordance to the model. The acquired adsorption statistics best fits with PSO. The R-squared result of linear plot represent the PSO is close to 1 for both the parameters.

Furthermore, the higher PSO R-squared clearly indicates that the adsorption mechanism is predominantly driven by chemical reactions. The acquired results support the findings of numerous researchers and show that the PSO is the best choice for investigating different adsorption processes^43–45^.

### Adsorption isotherm capacities

The Langmuir maximum adsorption capacities (qm) has a significantly higher for PWS adsorbent (3.7693 mg/g) compare to AC (2.2962 mg/g) and GM:ZEO (0.0372 mg/g). The qm values were obtained by intercepting the linear graph. The differences observed among these adsorbents due to increased cation exchange mechanism towards PWS adsorbent during ammonium adsorption compare to AC and GM:ZEO. The analysis of the Table 4 reveals that PWS has better adsorption isotherm capacities for both COD and NH_3_–N.

**Table 4:** Langmuir maximum adsorption capacity of the various adsorbents.

Adsorbent	Langmuir maximum adsorption capacities			References
	R^2^	${q}_{m}$	${K}_{L}$	
	Activated Carbon (AC)			^46^
COD	0.9975	2.2962	0.0002	
NH_3_–N	0.9454	0.7351	0.0012	
	Green mussel and Zeolite (GM:ZEO)			^27^
COD	0.9998	0.0372	0.0006	
NH_3_–N	0.9849	0.0083	0.0032	
	PWS			Present study
COD	0.9949	3.7693	0.7073	
NH_3_–N	0.9855	0.8985	0.5740	

## Conclusion

In conclusion, this investigation determined that 2.0 g per 100 ml of PWS performed best as an adsorbent. This ratio produced noticeably better results of eliminating COD and NH_3_–N compare to utilizing the adsorbent alone. However, using PWS has offers a lot of benefits, including decreased handling cost, cost effectiveness, and a decrease in waste disposal in landfills. In this study, a batch adsorption experiment was conducted using precise parameters, including pH7, 200 rpm stirring speed, 2.0 g per 100 ml dosage and 120 min of shaking time. The contact angle analysis demonstrated that the contact angle of PWS were 92.60° respectively, indicated that PWS were hydrophobic materials.

The maximum reduction efficiencies for COD and NH_3_–N at 2.0 g per 100 ml of PWS were measured 62% and 52%. Thus, 200 rpm of stirring was shown to be the best option for elimination percentages of 45% for COD and 42% for NH_3_–N. A shaking period of 120 min was obtained as the best removal percentage values of 48% and 42% for COD and NH_3_–N. The optimal removals for COD and NH_3_–N at pH 7 were measured 48% and 40%.

The investigation revealed that Langmuir and Freundlich models demonstrated the efficacy of this material removed the contaminant present in landfill leachate. According to the experiment results and the higher R-squared value, the Langmuir suited the data more closely than the Freundlich. The R-square for the Langmuir were 0.9949 for COD and 0.9919 for NH_3_–N representing a significant relationship between model predictions and experimental data. The lower R-squared values for the PFO indicate that the COD and NH_3_–N adsorption are not consistent with the assumptions and reactions of models. The R-square for PSO were very close to 1 for both COD and NH_3_–N with of 0.9987 and 0.9909 respectively. This study findings conclude that PWS is more effective as an adsorbent, indicating that it has the potential to replace more traditional adsorbents. Moreover, PWS seems to be suitable option for mitigating environmental contamination resulting from COD and NH_3_–N in leachate.

## Data Availability

The datasets used and/or analyzed during the current study available from the corresponding author on reasonable request.
